# Rapid Prediction of Bacterial Heterotrophic Fluxomics Using Machine Learning and Constraint Programming

**DOI:** 10.1371/journal.pcbi.1004838

**Published:** 2016-04-19

**Authors:** Stephen Gang Wu, Yuxuan Wang, Wu Jiang, Tolutola Oyetunde, Ruilian Yao, Xuehong Zhang, Kazuyuki Shimizu, Yinjie J. Tang, Forrest Sheng Bao

**Affiliations:** 1 Department of Energy, Environmental and Chemical Engineering, Washington University in St. Louis, St. Louis, Missouri, United States of America; 2 Department of Computer Science and Engineering, Ohio State University, Columbus, Ohio, United States of America; 3 Boxed Wholesale, Edison, New Jersey, United States of America; 4 State Key Laboratory of Microbial Metabolism, School of Life Sciences and Biotechnology, Shanghai Jiao Tong University, People’s Republic of China; 5 Institute of Advanced Biosciences, Keio University, Tsuruoka, Yamagata, Japan; 6 Department of Electrical and Computer Engineering, University of Akron, Akron, Ohio, United States of America; Hellas, GREECE

## Abstract

^13^C metabolic flux analysis (^13^C-MFA) has been widely used to measure *in vivo* enzyme reaction rates (i.e., metabolic flux) in microorganisms. Mining the relationship between environmental and genetic factors and metabolic fluxes hidden in existing fluxomic data will lead to predictive models that can significantly accelerate flux quantification. In this paper, we present a web-based platform MFlux (http://mflux.org) that predicts the bacterial central metabolism via machine learning, leveraging data from approximately 100 ^13^C-MFA papers on heterotrophic bacterial metabolisms. Three machine learning methods, namely Support Vector Machine (SVM), k-Nearest Neighbors (k-NN), and Decision Tree, were employed to study the sophisticated relationship between influential factors and metabolic fluxes. We performed a grid search of the best parameter set for each algorithm and verified their performance through 10-fold cross validations. SVM yields the highest accuracy among all three algorithms. Further, we employed quadratic programming to adjust flux profiles to satisfy stoichiometric constraints. Multiple case studies have shown that MFlux can reasonably predict fluxomes as a function of bacterial species, substrate types, growth rate, oxygen conditions, and cultivation methods. Due to the interest of studying model organism under particular carbon sources, bias of fluxome in the dataset may limit the applicability of machine learning models. This problem can be resolved after more papers on ^13^C-MFA are published for non-model species.

## Author Summary

Metabolic information is important for disease treatment, bioprocess optimization, environmental remediation, biogeochemical cycle regulation, and our understanding of life’s origin and evolution. ^13^C-MFA can quantify microbial physiology at the level of metabolic reaction rates. To speed up microbial characterizations and fluxomic studies, we hypothesize that genetic and environmental factors generate specific fluxome patterns that can be recognized by machine learning. Aided by constraint programming and quadratic optimization, our platform based on machine learning (ML) can predict meaningful metabolic information about bacterial species in their environments. Further, it can offer constraints to improve the accuracy of flux balance analysis. This study infers that the bacterial metabolic network has a certain degree of rigidity in allocating carbon fluxes, and different microbial species may share common regulatory strategies for balancing carbon and energy metabolisms. As a proof of concept, we demonstrate that the use of data-driven artificial intelligence (AI) approaches, e.g., ML, may assist mechanistic based models to elucidate the topology of microbial fluxomes.

## Introduction

With the advent of systems biology tools, such as genomics, transcriptomics, proteomics, and metabolomics during the last decade, the understanding of intracellular metabolisms from genotype to phenotype has been dramatically boosted. Notably, ^13^C metabolic flux analysis (^13^C-MFA) enables the quantification of metabolic reaction rates *in vivo* [1]. It determines carbon metabolic fluxes using the mass isotopomer distribution (MID) of proteinogenic amino acids or free metabolites from ^13^C labeling experiments. ^13^C-MFA is considered as a reliable measurement of central metabolic reaction rates [2], which has demonstrated its power in discovering novel pathways [3, 4], validating gene functions [3], verifying engineered strains [5, 6], and revealing energy metabolism of host strains [7]. In the past decade, advanced parallel bioreactor systems, mass spectrometry, and computational tools resolving metabolic fluxes have been developed [8–11], which improved the precision of flux profiles [12] and extended ^13^C-MFA’s application to the non-stationary metabolic phase [13, 14]. On the other hand, broad applications of ^13^C-MFA are still hindered because ^13^C experiments, biomass analysis, and flux calculations are expensive and time-consuming [15]. Moreover, some microbial systems may not be amenable to ^13^C-MFA if they require complex nutrients or their genome annotation is incomplete [16]. Before performing ^13^C-MFA on non-model species, laborious work is needed to examine extracellular metabolites, to characterize unknown pathways, and to analyze biomass compositions.

This study aims to employ an artificial intelligence (AI) approach called machine learning (ML) to investigate bacterial fluxomics patterns. ML is a powerful tool in systems biology [17] and has demonstrated successes in omics studies [18, 19]. For example, the precision of genome annotation on the model species *C. elegans* has been significantly enhanced by employing a simplified Support Vector Machine (SVM) method. Researchers have reached an accuracy of 75% on controversial genes [20]. At the transcriptomics level, ML approaches have been frequently used for disease identification. For instance, SVM has successfully recognized the gene expression patterns of hepatocellular carcinoma (HCC) [21], diffuse large B-cell lymphoma (DLBCL) [22] and ovarian cancer [23]. At the proteomics level, Supek *et al.* have employed a combined approach by integrating the Principal Component Analysis (PCA) method with SVM, to enhance analytic power in identifying “fingerprint” proteins (i.e., unique proteins in each tissue) from different horseradish tissues (leaf, teratoma, and tumor) grown *in vitro* [24]. In metabolomics, an SVM method can resolve the NMR data of metabolites in urine samples from different groups of people (healthy vs. pneumonia) [25]. In metabolic modeling, Karp’s group have adopted ML algorithms to predict the existence of various pathways for metabolic network reconstruction in different organisms [19].

The general idea of ML is to statistically build a numerical predictive *model* or an *estimator* which is a function *f* : ***X*** ↦ ***y*** that maps a vector of numbers called the *feature vector* to a vector of numbers called the *target* or the *label*. In many cases, the target is a 1-D vector, or a scalar. One may consider the feature vector as the input and the target as the output of the model. If the target takes discrete values, we call the ML model a *classifier*. Otherwise, a *regressor*. A commonly used classifier is binary classifier, where the cardinality (size) of the target set ***y*** is 2, e.g., ***y*** = {+1,−1}. In this paper, we build a regressor $f:R^{n}\mapsto R$ for each flux, where R stands for the set of all real numbers. In *supervised ML*, a pair of a feature vector and a target form a training *sample*. Given a finite set of *N* samples {(**X**_**1**_, *y*_1_), …, (**X**_**N**_, *y*_*N*_)}, an ML algorithm will find such a function, usually through solving a numerical optimization problem, to minimize the predictive error. Samples used to train a model form the *training set* while those for testing the performance form the *test set*. Given a new piece of data, numerically represented as a vector **X**_*new*_, the model *f* will predict the target *f*(**X**_*new*_), e.g., a flux value given reaction parameters where are represented by the vector **X**_*new*_ in this paper. The models learned through ML are usually not analytical models that can be represented using equations. Rather, they are numerical operators. For example, an artificial neural network (ANN) model can be represented by a series of weight and bias matrices, each of which is for one layer. A poor model can only predict well on the training set as if it only “remembers” the training samples, while a good model can learn the patterns among data and still be accurate on samples it has never “seen”. Hence, researchers make the training and test sets mutually exclusive. A mechanism called *cross validation* is used to ensure the mutual exclusiveness of training and test sets while making full use of the dataset.

A distinct advantage for ML applications is that they can reduce the need for costly experimental supplies and time-consuming benchwork. Despite the progress in utilizing ML methods in systems biology, there is no similar application in the fluxomics field to predict the flux profile. Therefore, we conceived the idea of integrating ML strategies with fluxomics research. To efficiently employ ML methods, a dataset with a sufficient number of samples is a prerequisite. Recently, a ^13^C-MFA dataset named CeCaFDB has been constructed, which includes more than 100 papers mostly on prokaryotic species [26]. Based on this dataset, five categorical and sixteen continuous features were initiated to describe the environmental and genetic factors involved in ^13^C-MFA of bacterial species. Unlike most omics projects employing ML approaches, this work built regressors rather than classifiers: 29 lumped central metabolic fluxes were adopted as the outputs to describe the central carbon metabolism of bacteria species. A 10-fold cross validation evaluated the performance of different algorithms. Furthermore, we included a knowledge-based system to check whether user inputs were biologically meaningful. Lastly, quadratic programming was employed to adjust the fluxes predicted by ML to satisfy stoichiometric constraints. Our web-based platform MFlux provides reasonable predictions for central metabolic flux profiles on 30 bacteria species, and it can be accessed online at http://mflux.org along with the training data. Although our platform is still in the early phase, our trial to integrate AI approaches with mechanistic models will have broad impacts on both systems biology and metabolic engineering fields.

## Methods

### Data collection

The dataset used to build MFlux are constructed from the literature. The total uptake rate of carbon sources is normalized as 100; all other fluxes are normalized based on the uptake rate of carbon sources. We obtained ^13^C-MFA information for bacterial species from the CeCaFDB dataset and added a few recent papers (approximately 120 papers in total, as of January 2015). ^13^C-MFA data related to photosynthetic bacteria was excluded in ML study because of their diverse CO2 fixation pathways, light-sensitive fluxomes, and insufficient sampling sizes for ML. For photosynthetic species, MFlux currently only reports a general description of their fluxomic features based on corresponding references.

In heterotrophic microorganisms, interconversions between glycolysis metabolites (phosphoenolpyruvate and pyruvate) and TCA cycle metabolites (oxaloacetate and malate) involve a set of anaplerotic reactions (e.g., phosphoenolpyruvate carboxylase, phosphoenolpyruvate carboxykinase, pyruvate carboxylase, and malic enzyme) serving as a key switch point for carbon flux distribution in bacteria [27]. These reactions balancing both carbon and cofactors may be employed by different microbial species. For example, *E. coli* anaplerotic pathways involve phosphoenolpyruvate carboxylase and malic enzyme, while *Bacillus* species furnish pyruvate carboxylase (the pyruvate shunt). In the case of *Corynebacterium*, both phosphoenolpyruvate carboxylase and pyruvate carboxylase are functional [28, 29]. These anaplerotic pathways can re-route fluxes when central pathway such as pyruvate kinase is knocked out. To ease the ML efforts, the anaplerotic pathways were lumped into two routes that exchanges fluxes between the TCA cycle and the glycolysis nodes: (*MAL* → *PYR* + *CO*_2_ and *PEP* + *CO*_2_ → *OAA*). This simplification also considered the fact that ^13^C-MFA has poor resolutions on anaplerotic fluxes because various combinations of these reactions could generate similar labeling patterns in amino acids [30].

### Feature vector for ML

**Fig 1:**
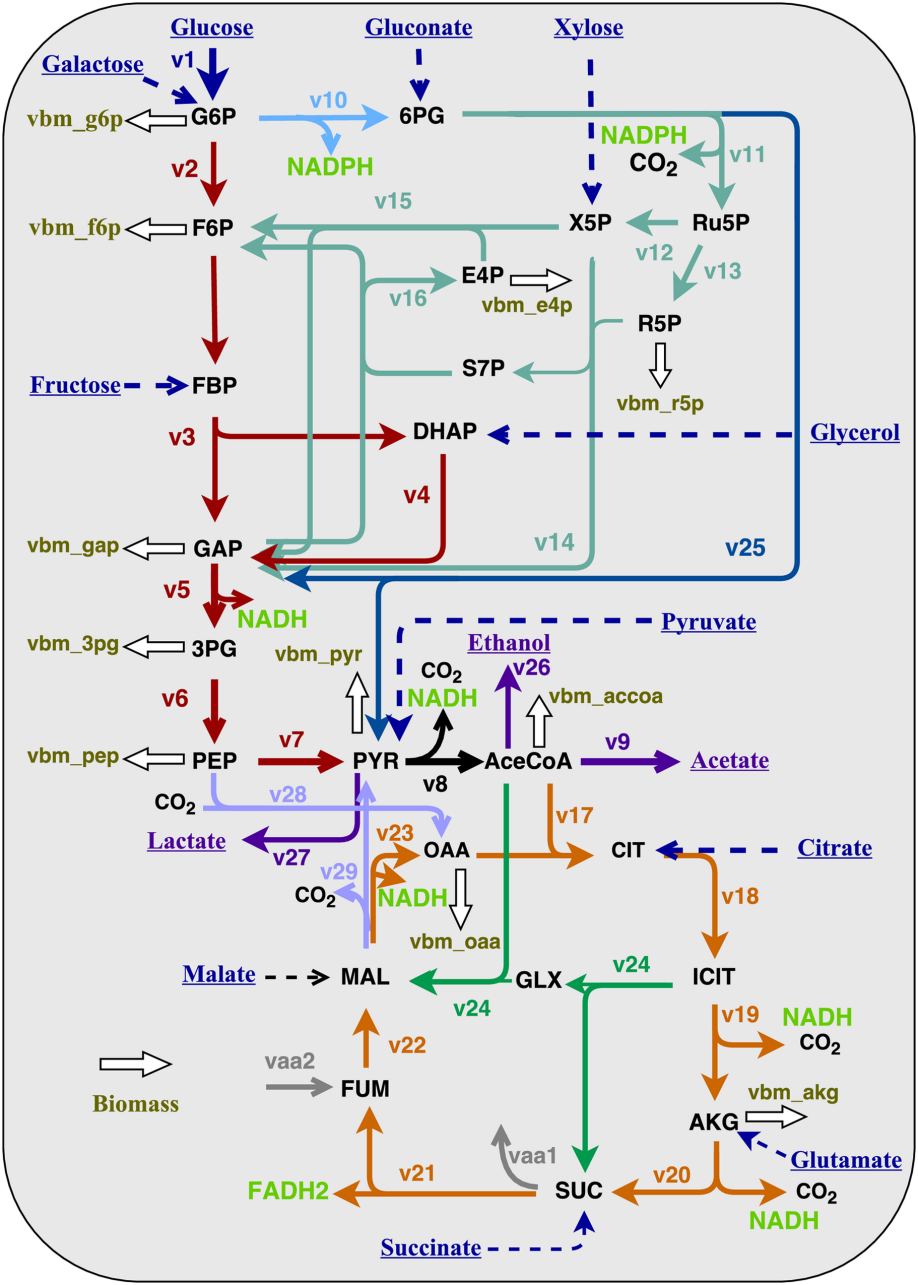
A universal central metabolic pathway for bacteria. The central carbon metabolic pathway is simplified into 29 fluxes, used as the outputs of our model.

As mentioned earlier, supervised ML builds models based on the samples, each of which is a pair of a feature vector and a target. Based on published ^13^C-MFA methodologies and microbial physiologies, we proposed five categorical features: species, nutrient types, oxygen conditions, engineering method, genetic background, and cultivation methods. There were two considerations when choosing those features. First, genetic modifications can significantly re-organize fluxomes. To improve the predictability on mutant strains, our platform allows toggling on or off certain central pathways (by manually setting the flux boundaries) in engineered strains. Second, the factor of cultivation method aims to reveal fluxome differences between shake flask cultures (a pseudo-steady state approach) and bioreactor cultures (a well-controlled fermentation or chemostat cultivation). Meanwhile, we introduced sixteen continuous features: growth rate, substrate uptake rate, and the ratio of multiple substrate uptakes (glucose, fructose, galactose, gluconate, glutamate, citrate, xylose, succinate, malate, lactate, pyruvate, glycerol, acetate and NaHCO_3_, as shown in Fig 1). Since the features include both categorical and continuous ones, *one-hot encoders* were used to convert categorical feature values into real numbers. Each feature was then standardized into zero mean and unit variance as assumed by many ML approaches. For each predicted flux, or the target/label in ML terminology, we scaled it into the interval [0, 1] by the min-max method. In addition to the min-max method, we also tested unit-variance-zero-mean standardization for scaling flux values, and the result was quite similar.

### Machine learning algorithms

The problem of predicting fluxes was modeled as a regression problem in ML where a computer program learns from existing data to estimate continuous variables. Twenty-nine regressors were trained to predict the 29 fluxes. We tested three widely-applied ML algorithms, including k-nearest neighbors (k-NN), decision tree, and SVM. To ensure a fair comparison, we performed a grid search for the best parameter set of each algorithm. The detailed parameter sets for 29 SVM-based regression models can be found from our web page. The programming language used for this project was Python 2.7 and the numpy and scikit-learn modules were utilized for machine learning [31]. Program files for training the models and testing them are wrapped in S1 Program. Full version including web-end code is released under GNU GPL v3 at https://bitbucket.org/forrestbao/influx

### Model evaluation and cross validation

Considering the limited number of samples in the current dataset, we adopted a 10-fold cross validation. An *N*-fold cross validation works as follows. All samples in our dataset are spliced into *N* equal parts. In each iteration, *N* − 1 parts are used as the training set, while the remaining as the test set. In the next iteration, the test set will be rotated to another part of the data, and the training set will consist of all other samples. This procedure will stop when all parts of the data have been incorporated into the test set exactly once, and training set exactly *N* − 1 times. Finally, the accuracy of the model can be calculated by checking the prediction result for each sample. For each flux, the error in cross validation is computed using Mean Squared Error (MSE).

### Stoichiometric constraints and boundary

One unique feature of our method is incorporating the overall mass balance through central metabolic pathways. The stoichiometric equations in Fig 1 under steady state are summarized as follows:

$$\text{G6P}:v_{1}=v_{2}+v_{10}+vbm_{g6p}$$ (1)

$$\text{F6P/FBP}:v_{2}+v_{15}+v_{16}+100\cdot ratio_{fructose}=vbm_{f6p}+v_{3}$$ (2)

$$\text{DHAP}:v_{3}+100\cdot ratio_{glycerol}=v_{4}$$ (3)

$$\text{GAP}:v_{3}+v_{4}+v_{14}+v_{15}+v_{25}=v_{5}+v_{16}+vbm_{gap}$$ (4)

$$\text{3PG}:v_{5}=v_{6}+vbm_{3pg}$$ (5)

$$\text{PEP}:v_{6}=v_{7}+v_{28}+vbm_{pep}$$ (6)

$$\text{PYR}:v_{7}+v_{25}+v_{29}+100\cdot ratio_{pyruvate}=v_{8}+v_{27}+vbm_{pyr}$$ (7)

$$\text{AceCoA}:v_{9}+v_{17}+v_{24}+v_{26}+vbm_{accoa}=v_{8}$$ (8)

$$\text{Ru5P}:v_{11}=v_{12}+v_{13}$$ (9)

$$\text{R5P}:v_{13}=v_{14}+vbm_{r5p}$$ (10)

$$\text{E4P}:v_{15}+vbm_{e4p}=v_{16}$$ (11)

$$\text{S7P}:v_{14}=v_{16}$$ (12)

$$\text{X5P}:v_{12}+100\cdot ratio_{xylose}=v_{14}+v_{15}$$ (13)

$$\text{6PG}:v_{10}+100\cdot ratio_{gluconate}=v_{11}+v_{25}$$ (14)

$$\text{CIT}:v_{17}+100\cdot ratio_{citrate}=v_{18}$$ (15)

$$\text{ICIT}:v_{18}=v_{19}+v_{24}$$ (16)

$$\text{AKG}:v_{19}+100\cdot ratio_{glutamate}=v_{20}+vbm_{akg}$$ (17)

$$\text{SUC}:v_{20}+v_{24}+100\cdot ratio_{succinate}=v_{21}+v_{aa1}$$ (18)

$$\text{FUM}:v_{21}+v_{aa2}=v_{22}$$ (19)

$$\text{MAL}:v_{22}+v_{24}+100\cdot ratio_{malate}=v_{23}+v_{29}$$ (20)

$$\text{OAA}:v_{23}+v_{28}=v_{17}+vbm_{oaa}$$ (21)

Specifically, *v*_1_ represents the flux from carbon substrate (either glucose or galactose) to G6P since both glucose and galactose can be catabolized to G6P, *vaa*_1_ and *vaa*_2_ represent fluxes involved in biomass building block synthesis or extracellular products, while *vbm* represents carbon fluxes going to biomass from different precursors [32].

A series of linear constraints can be derived from the stoichiometric equations above and used to restrain fluxes predicted by the ML methods:

$$v_{1}-100\cdot \left(ratio_{glucose}+ratio_{galactose}\right)=0$$ (22)

$$v_{3}-v_{4}+100\cdot ratio_{glycerol}=0$$ (23)

$$v_{11}-v_{12}-v_{13}=0$$ (24)

$$v_{14}-v_{16}=0$$ (25)

$$v_{10}-v_{11}-v_{25}+100\cdot ratio_{gluconate}=0$$ (26)

$$-v_{17}+v_{18}-100\cdot ratio_{citrate}=0$$ (27)

$$-v_{12}+v_{14}+v_{15}-100\cdot ratio_{xylose}=0$$ (28)

$$-v_{18}+v_{19}+v_{24}=0$$ (29)

$$-v_{22}+v_{23}-v_{24}+v_{29}-100\cdot ratio_{malate}=0$$ (30)

Among equations listed above, Eq 22 indicates the case for co-metabolism of both C6 sugars. Meanwhile, a list of inequality constraints can be drawn, given that all biomass fluxes are non-negative:

$$v_{1}-v_{2}-v_{10}\geq 0$$ (31)

$$v_{2}-v_{3}+v_{15}+v_{16}+100\cdot ratio_{fructose}\geq 0$$ (32)

$$v_{3}+v_{4}-v_{5}+v_{14}+v_{15}-v_{16}+v_{25}\geq 0$$ (33)

$$v_{5}-v_{6}\geq 0$$ (34)

$$v_{6}-v_{7}-v_{28}\geq 0$$ (35)

$$v_{7}-v_{8}+v_{25}-v_{27}+v_{29}+100\cdot ratio_{pyruvate}\geq 0$$ (36)

$$v_{8}-v_{9}-v_{17}-v_{24}-v_{26}\geq 0$$ (37)

$$v_{13}-v_{14}\geq 0$$ (38)

$$-v_{15}+v_{16}\geq 0$$ (39)

$$v_{19}-v_{20}+100\cdot ratio_{glutamate}\geq 0$$ (40)

$$-v_{17}+v_{23}+v_{28}\geq 0$$ (41)

$$-v_{21}+v_{22}\geq 0$$ (42)

Among all inequality constraints, Eq 39 works well except for the case of *zwf* knockout, where the direction of Eq 39 could be reversed [33].

### Flux adjustment using stoichiometric constraints

We adopted a quadratic programming method similar to minimization of metabolic adjustment (MOMA) [34], to tune fluxes to satisfy the stoichiometric constraints. The CVXOPT package for Python was employed here for quadratic programming [35]. The optimization problem is modeled as

$$\begin{array}{cc} \text{Minimize}\;\;f(v) & =\sum_{i=1}^{29}{\left(\text{Scaled}\left(v_{i}\right)-\text{Scaled}\left({\hat{v}}_{i}\right)\right)}^{2}\\ \text{Subject}\;\text{to}\;\;S\cdot v & =0,\\ A\cdot v & \geq 0, \end{array}$$ (43)

where the vector $\hat{v}=\left[{\hat{v}}_{1},\ldots ,{\hat{v}}_{29}\right]$ is the flux values predicted by ML, the vector **v** = [*v*_1_, …, *v*_29_] is the flux values to be solved in this optimization problem, the function Scaled(⋅) using Min-Max scaling to scale all fluxes into the range [0, 1], the matrix **S** is obtained from all equality constraints from Eq 22 to Eq 30, and the matrix **A** is obtained from all inequality constraints from Eq 31 to Eq 42. Notably, the biomass composition for a same species varies significantly under various conditions. Therefore, the quadratic programming looses mass balance constraints toward biomass synthesis. The purpose of scaling fluxes into the same range is to avoid the bias because fluxes have different dynamic ranges. The objective function *f*(**v**) can be rewritten into a standard quadratic programming problem using the following steps:

$$\begin{array}{cc} f(v) & =\sum_{i=1}^{29}{\left(\text{Scaled}\left(v_{i}\right)-\text{Scaled}\left({\hat{v}}_{i}\right)\right)}^{2}=\sum_{i=1}^{29}{\left(\frac{v_{i}-Min_{i}}{Max_{i}-Min_{i}}-\frac{{\hat{v}}_{i}-Min_{i}}{Max_{i}-Min_{i}}\right)}^{2}\\ & =2\cdot \sum_{i=1}^{29}\left(\frac{1}{2}\frac{v_{i}^{2}}{{\left(Max_{i}-Min_{i}\right)}^{2}}+\frac{-1\cdot v_{i}\cdot {\hat{v}}_{i}}{{\left(Max_{i}-Min_{i}\right)}^{2}}+\frac{1}{2}\frac{{\hat{v}}_{i}^{2}}{{\left(Max_{i}-Min_{i}\right)}^{2}}\right) \end{array}$$ (44)

where *Min*_*i*_ and *Max*_*i*_ are the range of the *i*-th flux. Since the last term $\frac{1}{2}{\left(\frac{{\hat{v}}_{i}}{Max_{i}-Min_{i}}\right)}^{2}$ and the coefficient 2 are constants, they can be omitted from the objective function. Hence, Eq 43 can be rewritten in standard quadratic programming form as

$$\begin{array}{cc} \text{Minimize}\;\;f(v) & =\frac{1}{2}\sum_{i=1}^{29}\frac{{\left(v_{i}\right)}^{2}}{{\left(Max_{i}-Min_{i}\right)}^{2}}+\sum_{i=1}^{29}\frac{-1\cdot v_{i}\cdot {\hat{v}}_{i}}{{\left(Max_{i}-Min_{i}\right)}^{2}}\\ \text{Subject}\;\text{to}\;\;S\cdot v & =0,\\ A\cdot v & \geq 0. \end{array}$$ (45)

For the upper and lower boundaries of each flux, i.e., *Max*_*i*_ and *Min*_*i*_, we used the maximal and minimal values observed in multiple datasets as the default values. Users can manually set desired values for the upper/lower bound of any specific flux in MFlux webpage, or they can opt to not use any boundaries. For instance, users can simply set the boundary of a certain flux as zero if this specific gene is knocked out.

### Constraint programming and input checking

To ensure user inputs, e.g., growth rates, oxygen usage, and substrate uptake rates, are biologically meaningful, MFlux first checks the satisfiability (e.g., whether cell growth rate is realistic) of input values [36]. The biological meaningfulness is represented using constraint programming [37], where each input is treated as a variable of a given domain. A set of inputs lacking of biological meaning will cause those constraints to be unsatisfied and MFlux will report an error message to warn the user. The Python module python-constraint [38] is used as the constraint solver.

### Overall system design

**Fig 2:**
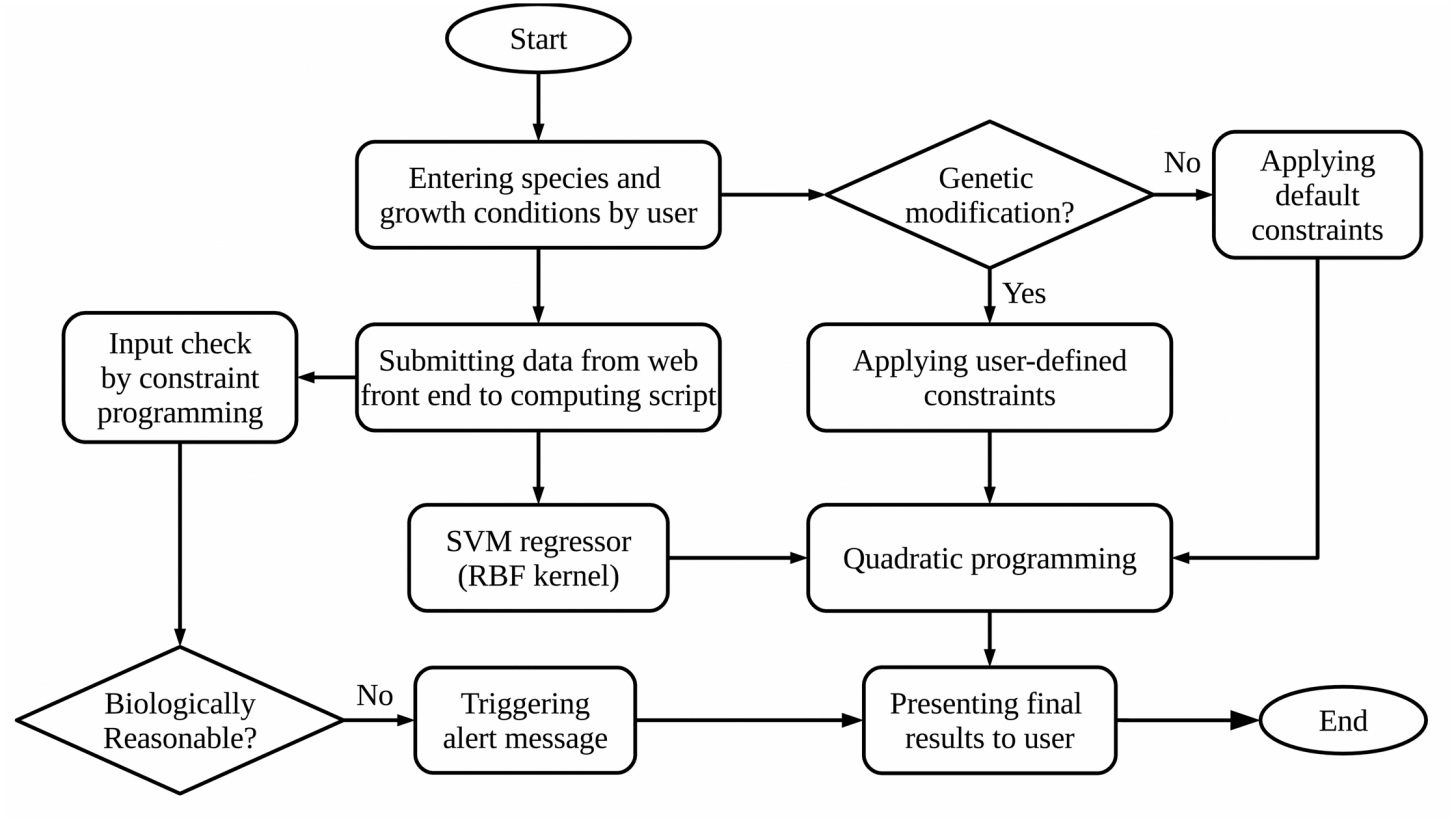
The flowchart of MFlux algorithm.

Different parts of MFlux mentioned above are put together as illustrated in Fig 2. The prediction on 29 fluxes is done via an RBF-kernel SVM, whose outcome will be finalized by quadratic programming. Users can set boundary constraints to represent information about genes that are knocked out on the species, and such information will be used in quadratic programming. If parameters set by the user are not biologically meaningful, a warning message will be displayed. In the future, users will also have the option to enter flux constraints and settings of their own experiment to improve the prediction accuracy of MFlux.

## Results

### Pathway map and statistical analysis

The core metabolism of bacteria is summarized into a pathway map in Fig 1. Considering the availability of information, 29 major fluxes with 14 potential substrates were used to represent a universal heterotrophic carbon metabolism for non-photosynthetic bacteria species, which includes glycolysis, the tricarboxylic acid (TCA) cycle, the pentose phosphate (PP) pathway, the Entner–Doudoroff (ED) pathway, the glyoxylate shunt and the anaplerotic pathway. It is difficult for ^13^C-MFA to precisely resolve the anaplerotic pathway fluxes [39]. Information on the anaplerotic pathway is either incomplete or not precise in many publications in our dataset. Consequently, we simplified the anaplerotic pathway into two reversible fluxes. Similarly, we ignored several overflow fluxes which occasionally appear in ^13^C-MFA anaerobic metabolisms (e.g., the secretion of formate, butyrate, or pyruvate), because of lacking sufficient samples for machine learning. The omission of those fluxes can also partially explain the high prediction error in some fluxes (e.g., *v*_8_: Pyruvate → Acetyl-CoA).

**Fig 3:**
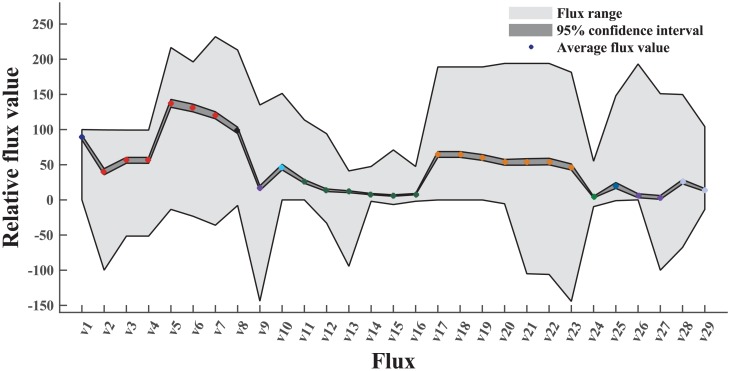
Overview of central metabolic fluxes collected in our dataset. “Flux range” represents the variation of each flux in the ^13^C-MFA dataset. “95% confidence interval” indicates that 95% of flux data were within a small range. “Average flux value” is the average value in each flux based on all data in our ^13^C-MFA dataset.

By statistical analysis, we determined the variation between each flux profile and the average flux profile from our ^13^C-MFA dataset. The average value, the range, and the 95% confidence interval for each flux are shown in Fig 3. The most conservative fluxes from our dataset include the non-oxidative pentose phosphate pathway and the glyoxylate shunt. The former pathway supplies precursors for bio-synthesizing amino acids (i.e., histidine, phenylalanine, and tyrosine) and nucleotides. The latter acts as an alternative carbon reserving path to the TCA cycle and is inhibited by the presence of glucose (most ^13^C-MFA is based on the glucose metabolism). All 29 fluxes are found to have a relatively narrow confidence interval compared to possible flux ranges, suggesting that fluxes of different bacteria species varies in a relatively small range. This is because most ^13^C-MFA studies are focusing on models species (e.g., *E. coli* and *B. subtilis*) and glucose based metabolism, while there are much less MFA efforts to study non-model species or metabolism of carbon substrates other than sugars (i.e., bias of fluxome research across).

### Optimization of algorithms and parameters

**Fig 4:**
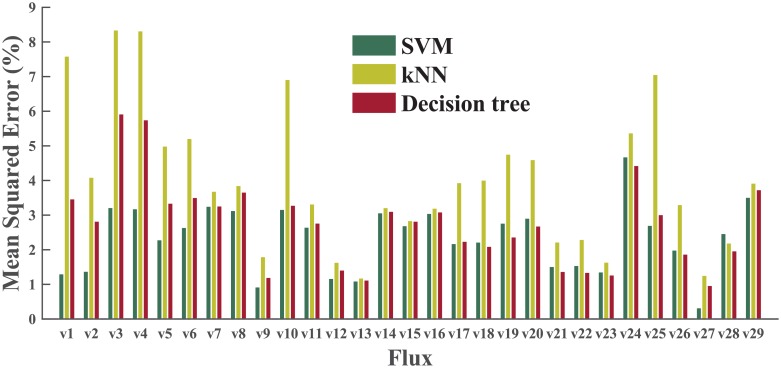
A comparison of three ML algorithms: SVM, k-NN, and decision tree. The best cross-validation results on 29 fluxes are compared. All tests in this step were performed on the WT dataset only.

To decide the most suitable ML algorithm, we first performed a grid search in the parameter space, using a dataset of wild type (WT) samples only. The best results of three different algorithms (for SVM, linear kernel only here) are presented in Fig 4. SVM makes better predictions than either the decision tree or k-NN on most fluxes. After this step, we carried out a second round of grid search to optimize parameters and improve the performance of SVM on the whole phenotype (WP) dataset (both WT and engineered). Both the linear kernel and radial bias function (RBF) kernel were included in this round of grid search.

**Fig 5:**
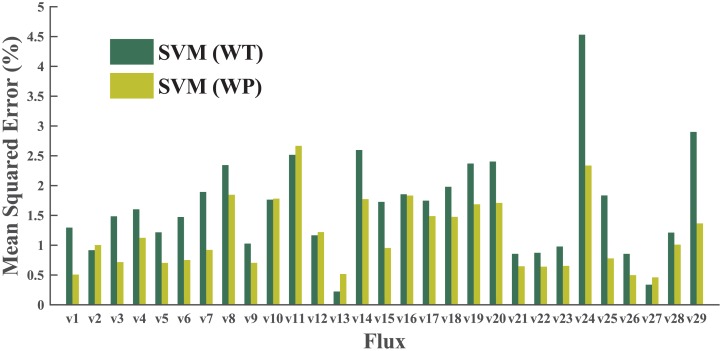
Best results by SVM on WT and WP datasets. Grid searches are performed on both linear and RBF kernels. The results from WP dataset are much better than those from the WT dataset. The result indicated that the size of the dataset is an important factor affecting the predictive power of machine learning models.

**Fig 6:**
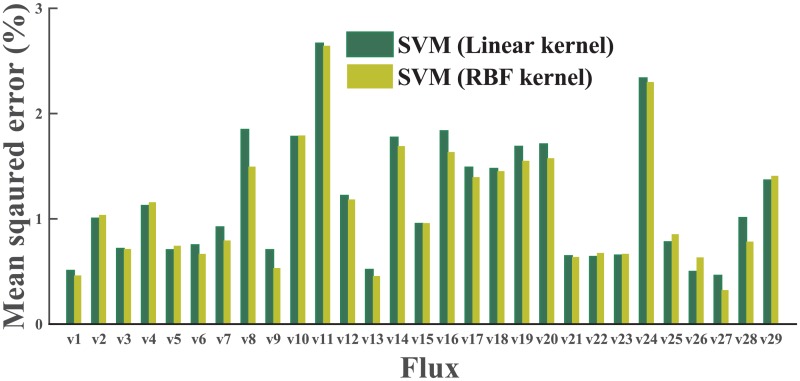
A comparison between linear-kernel SVM and RBF-kernel SVM. The best cross-validation results of linear kernel and RBF kernel after grid searches on WP dataset are very similar. The RBF kernel is employed in the final model for flux prediction.

Better cross validation was expected from the SVM models trained on the WT dataset, rather than on the WP dataset, while sophisticated genetic variations are not included in the WT dataset. However, cross-validation results refuted our initial thought: the models from the WP dataset demonstrated significantly better performance than those trained on the WT dataset (data shown in Fig 5). This result can be interpreted as that the size of the training set is a major factor affecting the model quality, especially when the training set is relatively small (the sizes of WT and WP datasets are about 150 and 450 samples, respectively). We also compared the SVM results using the linear kernel with those using the RBF kernel, and the RBF kernel showed slightly better performance (Fig 6). The parameter set producing the most accurate cross-validation result was used to configure MFlux. Notably, prediction on *v*_11_ (the second step of the oxidative PP pathway) and *v*_24_ (the glyoxylate shunt) have relatively large variations. Two factors may contribute to this fact. Both *v*_11_ and *v*_24_ have relatively narrow ranges (see Fig 1) and consequently even small numerical variations will generate larger relative errors for both fluxes. Meanwhile, genetic modifications may influence both *v*_11_ (e.g., *zwf* knockout [40]) and *v*_24_ (e.g., *ppc* knockout [41]) significantly. For instance, knocking out *zwf* in *E. coli* will cause a zero flux in *v*_10_ (the oxidative pentose phosphate pathway, OPP pathway) [42]. However, the lack of sufficient information on flux re-organization mechanisms in engineered microbes reduces ML predictability. This is because most engineered microbial fluxomics studies are focused on a few model species such as *E. coli*. To resolve this problem, the MFlux platform allows the users to manually set the boundaries of central fluxes to improve prediction quality (e.g., setting a zero flux through the OPP pathway for *E. coli zwf* mutant).

### Flux correction by quadratic programming

After parameter optimization, the SVM models of the best parameter sets can predict with relatively small error. However, the flux profile predicted by the ML method does not necessarily satisfy the inherent stoichiometric constraints of metabolic networks because the ML methods do have big enough dataset at this stage to reflect this. The situation could get even worse where specific fluxes predicted by the ML algorithm may go beyond a biologically meaningful range (e.g., the predicted glyoxylate shunt flux *v*_24_ may have a negative value). To address those issues, we employed quadratic programming for flux correction as described in the Methods section. More rational results with improved accuracy are expected after flux correction. An essential assumption of this step is that ML predictions are relatively close to real values reported in the literature. This hypothesis is backed by our cross-validation results further validated in the following case studies.

### Case studies

**Fig 7:**
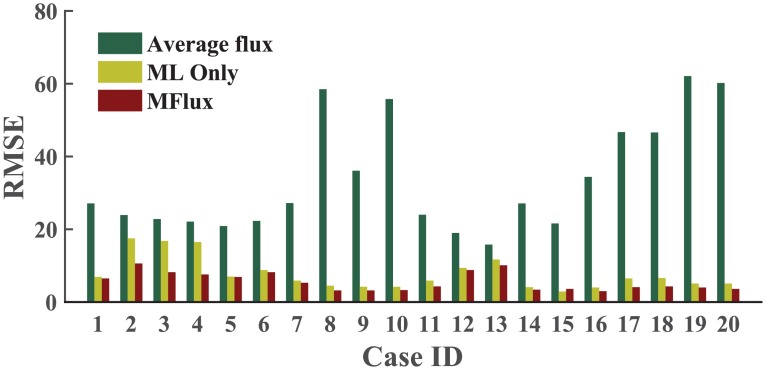
Summary of root mean squared error (RMSE) from 20 case studies: averaged flux from ^13^C-MFA dataset, ML-only, and MFlux (ML + quadratic programming). The average RMSE is 7.7 from ML-only, and 5.6 from MFlux. Detailed information is in S1 and S2 Tables.

To demonstrate the functionality of MFlux, we carried out tests on 20 cases, and the results are illustrated in Fig 7. Brief information for each case is listed in Table 1, and comprehensive results are included in S1 and S2 Tables. In general, MFlux can achieve decent flux predictions. Here we demonstrate two cases which are Cases 8 and 16.

**Table 1:** Summary of 20 cases of study. Glc, glucose; Xyl, xylose; Lac, lactate; Ace, acetate; KO, knockout.

Species	Carbon source	Oxygen condition	Reactor	Genetic background	Case
*E. coli*	Glc	aerobic	tube	WT	1 [12]
*E. coli*	Glc	aerobic	baffled shake flask	*ppc* KO	2–4 [41]
*B. subtilis*	Glc	aerobic	shake flask, CSTR	WT, *spo0A* KO	5–7 [43]
*B. subtilis*	Multiple	aerobic	shake flask	mutant	8–11 [44]
*C. glutamicum*	Glc	aerobic	shake flask	WT	12 [45]
*C. glutamicum*	Glc	aerobic	shake flask	mutant	13 [46]
*P. denitrificans*	Glc	aerobic, microaerobic	fermentor	WT	14, 15 [47]
*G. thermoglucosidasius*	Glc	microaerobic	shake flask	WT	16 [28]
*Thermoanaerobacter sp.*	Xyl	anaerobic	batch (closed)	WT	17, 18 [48]
*D. vulgaris*	Lac	anaerobic	batch (closed)	WT	19 [49]
*G. metallireducens*	Ace	anaerobic	batch (closed)	WT	20 [3]

**Fig 8:**
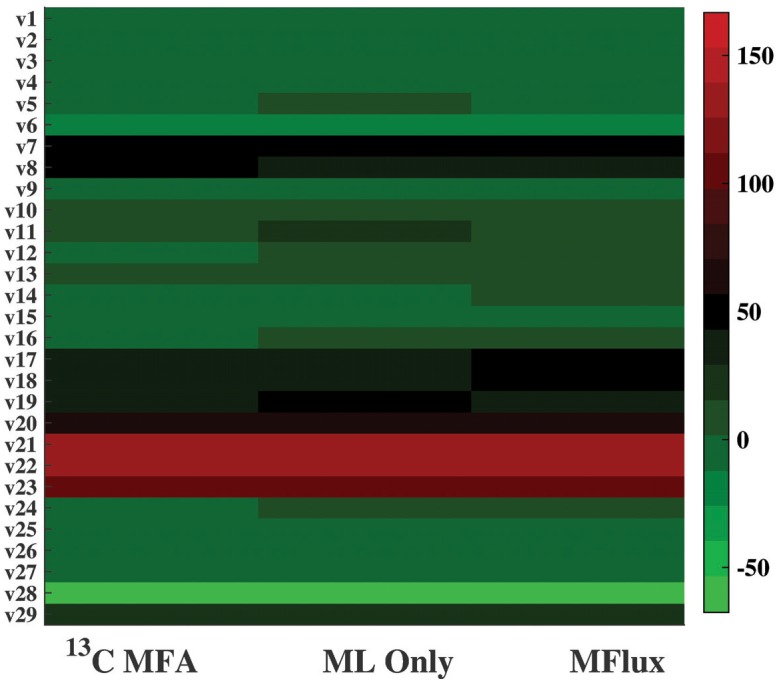
A comparison of the ^13^C-MFA flux, the flux predicted by ML only, and the flux predicted by MFlux in Case 8. *B. subtilis* was incubated in a shake flask (37 C, 300 rpm, aerobic condition), and supplied with labeled succinate and glutamate as carbon sources in M9 minimal medium. Detailed information is in S1 Table.

In Case 8, *B. subtilis* strain uptakes the mixed substrates succinate and glutamate. To illustrate mixed substrates co-metabolisms, we tested MFlux with ^13^C-MFA data of *B. subtilis* reported by Chubukov *et al*. [44]. Microbial fermentation fed with multiple substrates of low price is promising for the biotechnology industry. However, there are very few quantitative analyses of this topic. In this test, we adopted the same set of parameters found in the literature (S1 Table, Case 8) as the inputs of MFlux. For flux correction, we directly took the default boundary settings for quadratic programming. A comparison of flux profiles reported by ^13^C-MFA, predicted by ML only, and predicted by MFlux (i.e., ML + quadratic programming) is illustrated in Fig 8. ML-only approach and MFlux accurately predict on most fluxes, closely matching the ^13^C-MFA flux profiles with Root Mean Squared Error (RMSE) under 5. For ML, the predictions have large variation on specific fluxes (e.g., *v*_11_—oxidative PP pathway and *v*_19_– TCA cycle). Quadratic programming can further adjust flux profiles and reduce deviations of flux predictions. The corrected flux profiles also meet the basic stoichiometric relationship of the metabolic network. The final prediction from MFlux shows improvement with RMSE reduces to 3.2.

**Fig 9:**
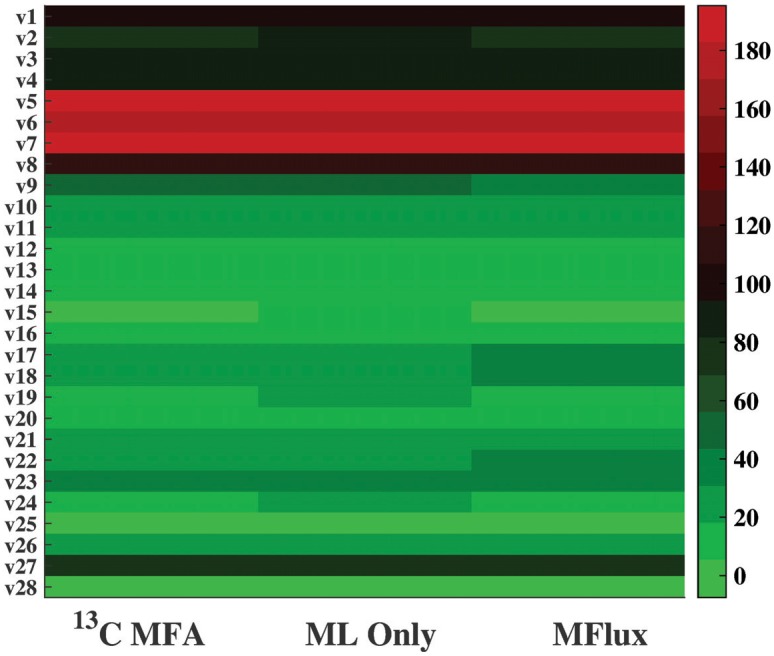
A comparison of the ^13^C-MFA flux, the flux predicted by ML only, and the flux predicted by MFlux in Case 16. *G. thermoglucosidasius* M10EXG was incubated in sealed bottles (micro-aerobic condition), supplied with glucose as a carbon source. Detailed information is in S2 Table.

In Case 16, *G. thermoglucosidasius* strain M10EXG grows under microaerobic conditions. *G. thermoglucosidasius* is a thermophilic and ethanol tolerant bacterium which can convert both hexose and pentose into ethanol [28]. To predict its central fluxomes, the parameter set used is listed in S1 Table, along with the default boundary settings for flux correction. A heat map (Fig 9) visualizes ^13^C-MFA fluxes with ML-only fluxes and MFlux results. The results are encouraging: ML-only prediction gives an RMSE of 4.0, while MFlux uses both ML and quadratic programming to improve the prediction to an RMSE of only 3.0. Among the 20 case studies, the average flux set has very large variations (RMSE of 33.5) from actual ^13^C-MFA fluxes (S2 Table). In this case, MFlux reduces the deviations of predicted fluxes from ^13^C-MFA values.

For species with genetic modifications in major pathways (Cases 2, 3, 4, 12, and 13, *E. coli* and *C. glutamicum*), MFlux predictions have an RMSE between 5 and 10, higher than the RMSE for prediction of wild type strains. Since MFlux is currently unable to capture complex regulatory mechanisms of flux reorganization, human-computer interaction can be employed by manually tuning boundary values of certain fluxes to improve flux prediction quality. For example, knocking out *ppc* on *E. coli* may activate the glyoxylate shunt [41, 42]. The users can assign a non-zero lower boundary of the glyoxylate shunt when running MFlux.

### Improving flux balance analysis of microbial metabolism via MFlux

**Fig 10:**
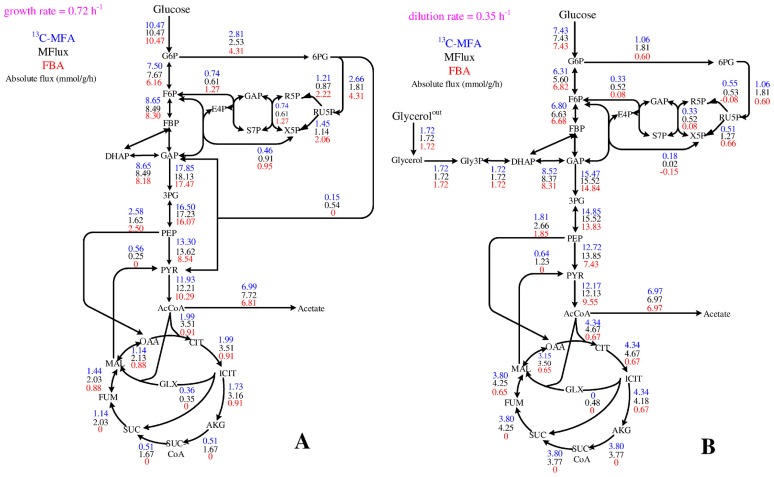
A comparison of the ^13^C-MFA flux, the flux predicted by MFlux, and the flux predicted by FBA. FBA analysis is simulated by an *E. coli i*JO1366 model (latest version) with default boundary settings from the reference [54]. The default values of growth associated maintenance energy (GAM) and non-growth associated maintenance energy (NGAM) were adopted. **A)** *E. coli* fluxome of glucose metabolism was precisely measured via parallel labeling experiments (a recent paper not in our dataset) [12]. **B)** *E. coli* fluxome of glycerol and glucose co-metabolism as measured by Drs. Yao and Shimizu (unpublished data). The *E. coli* strain was cultured in chemostat fermentor with a working volume of 1 L(37 C). The dilution rate in the continuous culture was 0.35 h^−1^. [1-^13^C] glucose and [1, 3-^13^C] glycerol were used for tracer experiments. The flux calculation is based on a previous method [42]. The RMSE from FBA is 22.5, while the RMSE from MFlux (this work) is 5.1. The COBRA toolbox running on MATLAB R2012b was employed for FBA/pFBA/geometricFBA simulation, and Gurobi 5.5 was used for linear programming. Detailed information is included in S2 Table.

Stoichiometry-based flux balance analysis (FBA) is an important mechanistic tool to predict unknown cell metabolism [50]. Accurate FBA prediction relies highly on setting the objective function and the flux constraints appropriately (based on thermodynamics or experimental analysis). Here, we compare FBA with MFlux for predicting *E. coli* metabolisms. The latest version of *E. coli i*JO1366 genome-scale model (2583 fluxes) was used [51]. Two comparative case studies were performed on *E. coli* fluxomes: one case for glucose based ^13^C-MFA via parallel labeling experiments [12] and the other for glucose and glycerol co-utilization (unpublished data from the Shimizu Group). Neither of the test cases was included in the training set of MFlux. Given ^13^C-MFA results as the control, MFlux results apparently have smaller RMSEs than FBA predictions. In the first case, the FBA has an RMSE of 11.3, while MFlux has an RMSE of 6.5 (Fig 10A). In the second case, the FBA has an RMSE of 22.5, while MFlux has an RMSE of 5.1 (Fig 10B). To circumvent variations caused by alternative solutions in FBA, we also employed pFBA and geometricFBA for both cases [52, 53] (S2 Table). In general, pFBA does not show better results compared with FBA for either case, while geometricFBA does not converge in our calculation.

FBA alone has given good predictions of growth rate as well as input and output fluxes, but not of intercellular fluxes. It is difficult to obtain actual P/O ratios, the ATP maintenance cost, the oxygen flux, and the transhydrogenase activities [55]. These energy/cofactor variables strongly affect the fluxes in the oxidative PP pathway (NADPH generation) and the TCA cycle (NADH, NADPH, and FADH_2_ generation). Without proper flux constraints and objective functions, it is more challenging for FBA to narrowly determine intracellular fluxomes in suboptimal metabolisms, especially for co-metabolism dual substrates because of the large solution space for the cell metabolism to optimize biomass growth using two substrates. As a complementary tool, MFlux may offer a quick metabolic overview and provide biologically meaningful flux boundaries to reduce FBA solution spaces when proper constraints for FBA are unavailable.

## Discussion

### Metabolic robustness of fluxome patterns among microbial species

“Robustness” was originally defined as the closed-loop process stability under perturbations in the control field. This definition is applicable to biochemical networks. To maintain the physiological output (i.e., the fluxome) within a desired range, microorganisms employ sophisticated control disciplines at different architecture levels, from the genome to the phenotype. In contrast to chaotic transcriptional profiles, the microbial fluxome shows robustness so that cells can survive in constantly-altering environments or in response to genetic mutations [56–58]. Metabolic rigidity at the flux level was first reported by Stephanopoulos in the early 1990s [59, 60]: NADPH is important for anabolism in the exponential growth phase, and the flux ratio around glucose-6-P node is rigid to form NADPH [60]. Moreover, 12 precursors from the central metabolism are required for biomass formation, which all have relatively small variations that are mainly dependent on biomass compositions. Due to both thermodynamic and mass balance constraints, cell metabolism aims to minimize variations in flux ratios under environmental perturbations. This rule also works for engineered microbes with moderately overexpressed pathways or strains from random mutations or deletions of non-essential genes. The feature of metabolic robustness facilitates ML applications.

Flux pattern recognition enables MFlux to predict metabolism of new species by learning from a small set of fluxome information from the same genus. For example, the metabolisms of *P. aeruginosa*, *P. fluorescens*, and *P. putida* have been studied by ^13^C-MFA in the past decade [61–65]. The results show that different *Pseudomonas* species employ remarkably identical fluxomics types: they employ a highly active ED pathway for glycolytic metabolism and keep a low flux on the PP pathway for biomass synthesis, due to the lack of the *pfk* gene [66]. The ED pathway has less cost for protein formation than the Embden–Meyerhof–Parnas (EMP) pathway, yet only one ATP is formed per glucose [67, 68]. *Pseudomonas* species have slow cell growth rates and their aerobic metabolisms do not yield by-products. They also demonstrate a very active pyruvate shunt (*MAL* → *PYR*) and NADPH overproduction flux (a benefit for counteracting oxidative stress). On the other hand, the TCA cycle in *Pseudomonas* species show plasticity under genetic and environmental variations [69], and can respond to increased ATP and NADH demands under stress conditions [70].

For different bacterial species (e.g., *E. coli* and *Bacillus*), their fluxomes (e.g., glucose metabolisms) can be similar, because central fluxes in catabolism are regulated by energy and building block requirements that show much smaller variations than genome or transcriptional differences. On the other hand, change of carbon substrates may alternate flux distributions. For example, co-utilization of glucose and glycerol in *E. coli* cause significant re-organization of fluxomes. In a same microbial strain, different fluxome patterns can be employed for metabolizing different substrates (e.g., glucose-based fluxome vs acetate based fluxomes). Recognizing these metabolic patterns allows the use of a relatively small training set to perform a decent metabolic prediction of diverse metabolic types. Consequently, these common principles of certain classes of microorganisms can be captured by machine learning for fluxome predictions.

### Limitations of machine learning

There are several major challenges regarding MFlux. First, the ^13^C-MFA flux in literature may have errors and biases, which would be included in the learning/training process of MFlux and lead to further variations. For example, current ^13^C-MFA studies are not evenly distributed among a broad scope of microbial genera. Most reported MFAs are concentrated in a few model microbial species or metabolism of only a few substrates (mainly glucose), and thus our current ML cannot predict fluxomes well in certain cases. Such problem (model bias) can be resolved after more ^13^C-MFA papers for non-model species are included in the database and more constraints are implemented by our platform.

Second, the predictability of ML is limited to species and pathways that are already included in learning. More information and efforts are required to deal with cases of genetically modified strains with engineered pathways that hijack flux for synthesis of diverse commodity chemicals [13]. Currently, ^13^C-MFA has not widely used by synthetic biology community yet. In future versions of MFlux, new metabolic knowledge and rules should be applied for flux corrections.

Third, it is still difficult to incorporate regulation mechanisms into the current model. For instance, various catabolite repression mechanisms regulate the cell fluxome in the presence of multiple substrates (e.g., glucose shows catabolite repression for fast growing *E. coli* when both glucose and glycerol are available, Fig 10) [71]. These hierarchy regulations among substrate utilization can be dependent on growth rates or can differ among microbial species (*E. coli*, *Bacillus* and *Corynebacterium*).

Fourth, when oxygen is not available, fast bacterial sugar utilization will activate mixed acid fermentation (e.g., by utilizing lactate dehydrogenase and pyruvate formate lyase) to produce complicated overflow metabolites [13]. This mechanism is also furnished in yeast and mammalian cells. However, ^13^C-MFA studies on anaerobic metabolisms are much less frequent than on aerobic metabolisms. MFlux cannot predict the complicated patterns of overflow fluxes at this stage.

Fifth, our current dataset is still unable to support ML studies on phototrophic bacterial fluxomes. For phototrophic metabolism, its energy generation (ATP, NADH and NADPH) may not be controlled by substrate catabolism. Some phototrophic bacteria (e.g., cyanobacteria) have versatile autotrophic and photomixotrophic metabolism that is highly sensitive to light and substrate availability. Other phototrophs may even have *CO*_2_ fixation pathway (such as the reversed TCA cycle). Therefore, our MFlux platform could not make ML predictions but only reports a general description of metabolic features of these species.

Lastly, ML cannot directly estimate fluxes for carbon sources which are not part of the learning dataset. To predict fluxomes for new substrates, users need to assume that similar entry-points of carbon sources into the central metabolic network may cause similar flux distributions (e.g., sucrose has to be treated as a combination of glucose and fructose).

### Conclusion

This proof-of-concept study demonstrates that AI methods can facilitate fluxomics research with reasonable precision. ^13^C-MFA is a very small field of just hundreds of MFA research papers on microbial species published in the past two decades. In the long term, ML methods may solve this problem: with a large and reliable fluxomics dataset and more information from ^13^C-MFA and AI scientists, the future MFlux model can make broad-scope metabolism predictions. To sum up, MFlux presents the first platform introducing ML in the field of fluxomics and it will be continuously updated and improved. It will inspire the development of similar computational tools to advance omics and metabolic engineering fields [72].

## Supporting Information

S1 Program MFlux Computer Program (Source code). Python scripts in a ZIP file.
(ZIP)

S1 Table Results of 20 case studies. Detailed information for 20 cases studies using MFlux, including literature references, input conditions, ^13^C-MFA flux, the flux profiles predicted by ML, and the flux profiles predicted by MFlux with additional constraints.
(XLSX)

S2 Table Detailed information of the comparison with FBA/pFBA. The information of constraints, objective function, and simulation results.
(XLSX)
